# Meropenem-resistant Burkholderia pseudomallei: a concerning single case in Australia with no prior meropenem exposure

**DOI:** 10.1099/acmi.0.000619.v4

**Published:** 2024-10-28

**Authors:** Pirathaban Sivabalan, Ferris Satyaputra, Ian Gassiep, Brian Forde, Jaimie Frazer, Matthew Glover, Buenafe Adams, Robert Norton

**Affiliations:** 1 Pathology Queensland, Townsville Hospital, Douglas, Townsville, Queensland, Australia; 2 University of Queensland, Brisbane, Queensland, Australia; 3 Department of Infectious Diseases, Mater Hospital Brisbane, South Brisbane, QLD 4101, Australia; 4 University of Queensland Centre for Clinical Research, Royal Brisbane and Woman’s Hospital, Herston, QLD 4029, Australia; 5 Pathology Queensland, Royal Brisbane and Women’s Hospital, Herston, QLD 4029, Australia

**Keywords:** *B. pseudomallei*, melioidosis, meropenem, resistance

## Abstract

We report a case of cutaneous melioidosis in a 54-year-old male with a meropenem-resistant sub-population. He was empirically treated with episodic doxycycline and trimethoprim–sulfamethoxazole; however, the abscess re-accumulated. The patient had no prior exposure to meropenem. A sub-population of the isolate was meropenem resistant with an MIC >32 µg ml^−1^ and the identification was re-confirmed as *Burkholderia pseudomallei*. Whole-genome sequencing with ARDaP analysis only revealed a resistance determinant to doxycycline and did not reveal a resistance determinant to meropenem. Furthermore, no carbapenemases were detected through multiple bioinformatics tools. To date, this is the first reported case in Australia of a *B. pseudomallei* isolate resistant to meropenem without previous carbapenem exposure.

## Data summary

This case reveals a sub-population of a *B. pseudomallei* isolate that was meropenem resistant with an MIC >32 µg ml^−1^, and the identification was re-confirmed as *B. pseudomallei*. WGS with ARDaP analysis only revealed a resistance determinant to doxycycline and did not reveal a resistance determinant to meropenem. Furthermore, no carbapenemases were detected through multiple bioinformatics tools. The raw sequence reads of the monoisolate (*B. pseudomallei* strain:TSV-377/isolate:TSV-377) can be found on the BioProject with the accession number PRJNA1032635, ID: 1 032 635 and locus tag prefix of R6D44 [1]. WGS data for TSV-377 has been submitted to the SRA under accession number SRR26538081.

## Introduction

*Burkholderia pseudomallei* is a bipolar Gram-negative bacillus found predominantly in the soil of tropical and subtropical regions of the world. The organism is the causative agent of melioidosis, a clinical disease with significant morbidity and mortality. It has multiple clinical presentations and complications including bacteraemia. The estimated case fatality rate of this disease is 15–42 %, which highlights the significance of appropriate treatment of this fatal disease [2,3].

The treatment generally involves a two-stage process, with an intensive intravenous phase and an oral eradication phase. The intensive phase involves either intravenous ceftazidime or meropenem, usually with a duration of 2–8 weeks depending on the site of infection and complications. Infections involving the bone or the brain tend to require a longer duration of intravenous antimicrobial therapy. Eradication therapy involves trimethoprim–sulfamethoxazole, doxycycline or amoxicillin–clavulanic acid. The duration of eradication therapy usually varies between 3 and 6 months again depending on the site and complication of the infection [4]. *In vitro* data support the preferential use of carbapenems for severe infection. *In vitro* time-kill studies to measure the rate of bacterial killing have shown that the carbapenems perform better against *B. pseudomallei* than ceftazidime [5,7]. Hence, the development of carbapenem resistance would be concerning, given its importance in the critical care setting and the limited antibiotics available to treat this potentially fatal infection. To date, naturally occurring resistance of *B. pseudomallei* to carbapenems is extremely rare [5].

We report a case of cutaneous melioidosis that describes the first clinical isolate of *B. pseudomallei* from Australia that exhibits meropenem resistance without prior exposure.

## Case report

**Fig. 1.**
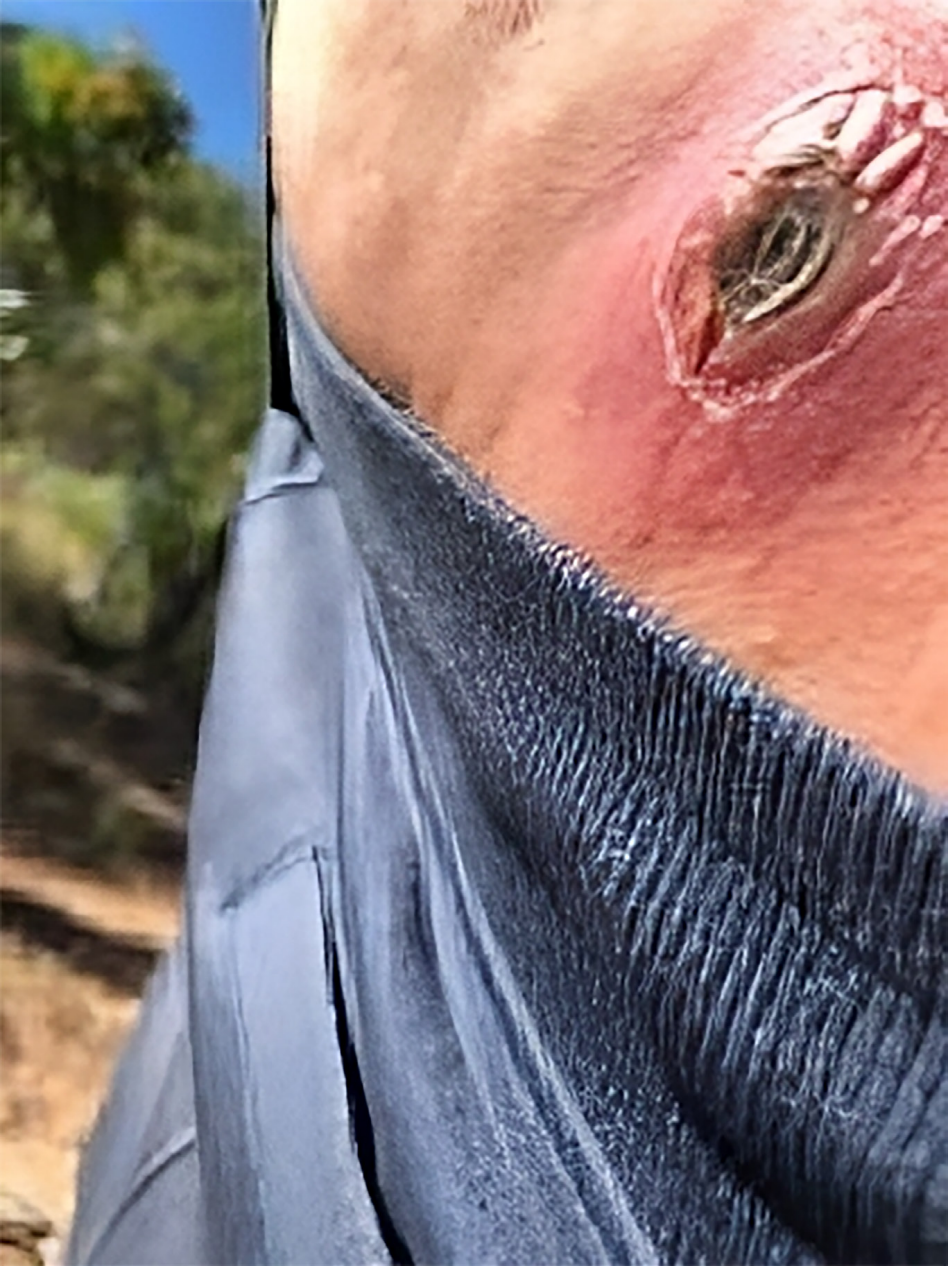
Cutaneous abscess of the right lower quadrant.

In November 2021, a 54-year-old male with type II diabetes mellitus, chronic kidney disease, polyarthropathy of unclear cause on regular prednisolone (12 mg daily) and metabolic syndrome presented to the hospital with multiple cutaneous abscesses on the right lower quadrant of the abdomen and right thigh. These had started on the abdominal wall (Fig. 1) in May 2021 after he had been walking through river water with open skin wounds. His local community doctor had prescribed multiple short courses of oral antibiotics, which included doxycycline and trimethoprim–sulfamethoxazole. The patient denied previous meropenem exposure. The lesions initially improved with trimethoprim–sulfamethoxazole and doxycycline; however, post-cessation, there was a re-accumulation of pus. Upon further history and examination, no systemic symptoms were identified. The patient underwent surgical debridement of the abdominal wall lesions. A surgical tissue specimen was processed in the microbiology laboratory and set up on horse blood agar (HBA) (both in 5% CO2 and anaerobic incubation conditions), MacConkey and crystal violet, colistin and nalidixc acid and chocolate agar (CHOC). Growth was monomicrobial and pure. The isolate was culture positive for *B. pseudomallei*.

**Fig. 2.**
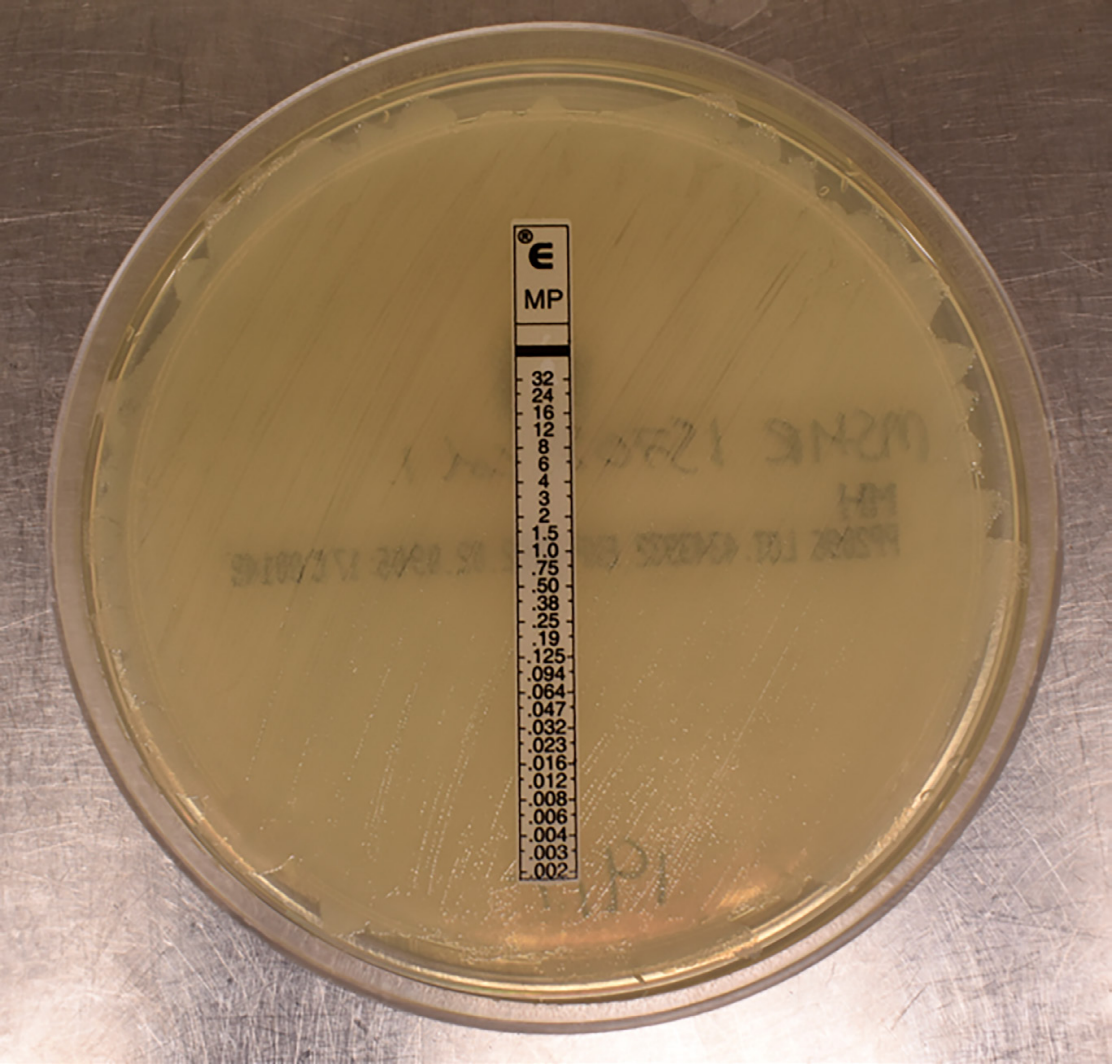
*B. pseudomallei* (isolate 2) with an MIC >32 µg ml^−1^.

The isolate was not identified on either the Vitek 2 or the API 20NE (bioMérieux, Marcy l’Etoile, France) despite repeated attempts with older growth at 48 h. *B. pseudomallei* identification was confirmed by molecular detection of the *TTS1* gene (a PCR assay used for the detection of *B. pseudomallei*) [8]. A single colony was taken from the HBA plate and antimicrobial susceptibility testing was performed as per the Clinical and Laboratory Standards Institute (CLSI) protocol [9], with meropenem (extrapolated from imipenem breakpoints), trimethoprim–sulfamethoxazole, doxycycline and ceftazidime Etest strips (bioMérieux) and incubated with 0.5 McFarland’s suspension of the isolate inoculated onto Muller–Hintonagar plates for 20 h in ambient air. Susceptibility testing revealed MICs of 1 µg ml^−1^ for ceftazidime (susceptible), >256 µg l^−1^ for doxycycline (resistant) and 1 µg ml^−1^ for trimethoprim–sulfamethoxazole (susceptible). The meropenem MIC was 4 µg ml^−1^; however, there were two c.f.u. within the ellipse of inhibition that appeared to have MICs of ≥4 µg ml^−1^. Given the rarity of this phenomenon in melioidosis, this was repeated again showing similar findings. Both isolates (meropenem susceptible and resistant isolates) were then sub-cultured onto separate HBA plates and re-incubated. Both isolates were confirmed as being *B. pseudomallei* by *TTS1* gene detection. Antimicrobial susceptibility testing was performed on both isolates. One isolate (*isolate 1*) revealed an MIC of 4 µg ml^−1^ for meropenem (susceptible), 1 µg l^−1^ for ceftazidime (susceptible), 0.25 µg ml^−1^ for trimethoprim–sulfamethoxazole (susceptible) and 16 µg ml^−1^ for doxycycline (resistant). The second isolate (*isolate 2*), however, revealed an MIC of >32 µg ml^−1^ for meropenem (resistant) (Fig. 2), 4 µg ml^−1^ for ceftazidime (susceptible), 0.5 µg ml^−1^ for trimethoprim–sulfamethoxazole (susceptible) and 64 µg ml^−1^ for doxycycline (resistant). Meropenem antimicrobial susceptibility testing was performed by different operators and in different laboratories, all with similar results highlighting the reproducibility of this result.

Given the high-level meropenem resistance of isolate 2, it was further assessed to exclude *Burkholderia ubonensis. B. ubonensis* belongs to the *Burkholderia cepacia* complex [10]. It can be commonly isolated from water and soil; however, it is rarely found in clinical isolates. It could be co-isolated with *B. pseudomallei,* especially in environmental samples. It can be easily confused with *B. pseudomallei*, as it has a similar morphotype on Ashdown’s media. However, to date, it is thought to be non-pathogenic [11]. * B. ubonensis* is commonly associated with high-level antibiotic resistance, including carbapenems. This is mediated by an inducible class A β-lactamase [10]. The concern surrounding identifying *B. ubonensis* is potential resistance transfer to other *Burkholderia* species [10]. Isolate 2 was analysed with a *B. ubonensis*- specific real-time PCR Assay Bu550, and it was negative. It is acknowledged that as the isolate was TTS1 positive by PCR, it would unlikely be *B. ubonensis*.

Isolate 2 underwent whole-genome sequencing (WGS), via MiniSeq System (Illumina Inc., San Diego, CA, USA), to help determine a mechanism of antimicrobial resistance. The isolate was identified as *B. pseudomallei* using the Kraken 2 (version 2.2.1) program. Genotypic resistance was assessed via Abricate (v1.0.1) using both the Comprehensive Antibiotic Resistance Database (CARD) and ResFinder database to look for the presence of carbapenemases. No carbapenemases were detected. The isolate was analysed with a bioinformatic tool [ARDaP (Antimicrobial Resistance Detection and Prediction)] designed to detect *B. pseudomallei* resistance [12]. The ARDaP database has been specifically designed for *B. pseudomallei*; thus, this is the most reliable database in regard to detecting genomic determinants of resistance. The only resistance determinant detected was BPSL3085 Ala88fs, which conferred resistance to doxycycline, which was also detected phenotypically in this isolate. No resistance determinants were detected for meropenem, ceftazidime, imipenem and trimethoprim–sulfamethoxazole. This result does not exclude the possibility of unknown resistance determinants.

## Discussion

We report a sub-population of *B. pseudomallei* in a clinical isolate resistant to meropenem, without prior meropenem exposure. Primary resistance of *B. pseudomallei* to carbapenems is extremely rare. A recent study looking a β-lactam susceptibility for * B. pseudomallei* isolates from Northeast Thailand by Fen *et al.* revealed that all 1317 primary isolates were imipenem susceptible. However, they observed two ceftazidime-resistant isolates, one ceftazidime intermediate isolate, two meropenem less-susceptible isolates and one amoxicillin– clavulanic acid resistant and two amoxicillin–clavulanic acid intermediate isolates [13]. This finding was also mirrored by the analysis of 234 isolates from the Northern Territory, Australia, with no resistance to carbapenems found [14].

To date, only a few case reports have described carbapenem resistance. Bugrysheva *et al.* performed genome sequencing on a * B. pseudomallei* strain isolated from the sputum of a hospital patient in the USA but believed to be acquired from Australia where the patient travelled several years before. This strain was reported to be resistant to imipenem, ceftazidime, amoxicillin–clavulanic acid, trimethoprim–sulfamethoxazole and doxycycline [15]. A case report by Nuryastuti *et al.* also described a meropenem-resistant strain isolated from an ulcer swab from the plantar aspect of the foot, following treatment with multiple antimicrobials including trimethoprim–sulfamethoxazole, clindamycin, cefixime and ciprofloxacin with no improvement [16]. The strain was identified based on the growth in Ashdown media, the characteristic Gram stain and the result of 16S rRNA, which confirmed *B. pseudomallei* [15]. However, resistance was detected using a disc diffusion method that was not a standard antimicrobial susceptibility testing method according to the CLSI [16].

Consequently, information regarding the underlying molecular mechanism of carbapenem resistance in *B. pseudomallei* is limited. Specific mutations (Thr147Ala) affecting the *PenA* gene located on chromosome 2 can lead to imipenem resistance [17]. Furthermore, Sarovich *et al.* highlighted the significant role of efflux pumps in mediating meropenem resistance [17]. They performed genomic analysis on *B. pseudomallei* isolates that developed reduced susceptibility to meropenem during treatment (MIC of 3–8 µg ml^−1^ from an initial MIC of 0.5–0.75 µg ml^−1^) and found that alteration of several genes that affect efflux pumps of the resistance–nodulation division (RND) family contributed to meropenem resistance. *AmrR* is the regulator gene of the RND efflux pump, AmrAB-OprA, and is responsible for intrinsic aminoglycoside and macrolide resistance in *B. pseudomallei*. Different mutations in the *amrR* gene were found to significantly upregulate AmrAB-OprA, increasing the meropenem MIC to 3 µg ml^−1^. The presence of sub-inhibitory meropenem concentration, which acts as a substrate for this efflux pump, could further induce upregulation up to 10- to 30-fold in isolates with *amrR* mutation. Mutations in other regulator genes can also be associated with reduced meropenem susceptibility. The other regulator genes involved are *bpeR*, a regulator of BpeAB-OprB RND efflux pumps, and *bpeT*, a regulator of BpeEF-OprC RND efflux pump.

Potential cross-resistance among antimicrobials used in melioidosis treatment was also demonstrated in our patient. Two strains of *B. pseudomallei* that had not been exposed to meropenem but had been exposed to doxycycline and trimethoprim–sulfamethoxazole showed reduced meropenem susceptibility. As doxycycline and trimethoprim–sulfamethoxazole are known substrates for *B. pseudomallei* RND efflux pumps [17], this raises the concern that these antibiotics precipitated the meropenem-resistance phenotype due to increased efflux pump activity. This finding that the development of antimicrobial resistance as a result of treatment with other classes of antibiotics is quite concerning, considering that this further restricts the already limited antimicrobial options. Unfortunately, sequencing analysis did not detect any resistance determinants for meropenem in our case. The most likely reason for this is a novel resistance mechanism that is not included in this database. Furthermore, if the mechanism of resistance was caused by an upregulation of an efflux pump, then this would be harder to detect using a DNA sequence study.

One limitation of this study is that it did not completely exclude the possibility of resistance that may have developed *in vitro* during susceptibility testing. Whilst developing resistance during antimicrobial susceptibility testing especially on initial exposure for 20 h would be rare, the possibility of this is not entirely excluded.

## Conclusion

Our case report provides an opportunity to review the available literature related to *B. pseudomallei* resistant to carbapenems. Given the limited antibiotic options to treat this potentially fatal infection, understanding the mechanisms of resistance is important to help prevent future resistance. This is especially important for meropenem, which is usually the standard of care for critically unwell patients requiring intensive care support. Further studies are essential to better elucidate the underlying mechanism of resistance in *B. pseudomallei,* particularly on the complex regulation of the efflux pumps including the role of any associated genes. Hopefully, in the era of sequencing, more knowledge will be acquired about antimicrobial resistance within *B. pseudomallei*.

## Abbreviations

CLSI: Clinical and Laboratory Standards Institute
HBA: horse blood agar
RND: resistance–nodulation division
WGS: whole-genome sequencing
